# 46,XX Differences of Sex Development outside congenital adrenal hyperplasia: pathogenesis, clinical aspects, puberty, sex hormone replacement therapy and fertility outcomes

**DOI:** 10.3389/fendo.2024.1402579

**Published:** 2024-05-22

**Authors:** Marianna Rita Stancampiano, Silvia Laura Carla Meroni, Carmen Bucolo, Gianni Russo

**Affiliations:** Department of Pediatrics, IRCCS San Raffaele Scientific Institute, Milan, Italy

**Keywords:** 46, XX DSD, gonadal differentiation, atypical genitalia, gonadal dysgenesis, ovotestis, Mayer-Rokitansky-Küster-Hauser syndrome, aromatase deficiency

## Abstract

The term ‘differences of sex development’ (DSD) refers to a group of congenital conditions that are associated with atypical development of chromosomal, gonadal, and/or anatomical sex. DSD in individuals with a 46,XX karyotype can occur due to fetal or postnatal exposure to elevated amount of androgens or maldevelopment of internal genitalia. Clinical phenotype could be quite variable and for this reason these conditions could be diagnosed at birth, in newborns with atypical genitalia, but also even later in life, due to progressive virilization during adolescence, or pubertal delay. Understand the physiological development and the molecular bases of gonadal and adrenal structures is crucial to determine the diagnosis and best management and treatment for these patients. The most common cause of DSD in 46,XX newborns is congenital adrenal hyperplasia (CAH) due to 21-hydroxylase deficiency, determining primary adrenal insufficiency and androgen excess. In this review we will focus on the other rare causes of 46,XX DSD, outside CAH, summarizing the most relevant data on genetic, clinical aspects, puberty and fertility outcomes of these rare diseases.

## 1 Introduction

Phenotypic sex is the result of a complex and multistep developmental process, controlled by different gene pathways and transcriptional factors, determining specific hormone secretion during critical stages of fetal life (1, 2). Sexual development involves two different developmental processes: sex determination (divided into chromosomal sex determination and gonadal sex determination) and sex differentiation (3, 4). Chromosomal sex determination already occurs during fertilization, when a sperm contributes either an X or Y chromosome to the X chromosome in the oocyte, determining an XX or XY zygote (5). Gonadal sex determination is a complex and dynamic multistep event, regulated by different genetic pathways, leading the bipotential indifferent gonad either into an ovary or a testis (4, 6). Sex differentiation occurs once the gonad has developed and is induced by the hormones produced by gonadal tissues, establishing the internal and external genitalia and a male or female phenotype (6–10).

Differences of sex development (DSD) are defined as congenital conditions with atypical development of chromosomal, gonadal, and/or anatomic sex (11). According to the Chicago Consensus (11), recently revised in a European Consensus Statement (12), DSD could be classified into chromosomal DSD, 46, XY DSD and 46,XX DSD. The 46,XX DSD group includes a wide spectrum of conditions due to atypical gonadal development and altered hormonal secretion; in the same group are also classified patients with atypical differentiation of Mullerian structures, affected by the Mayer-Rokitansky-Kuster-Hauser syndrome (MRKH).

The impact of DSD diagnosis in the affected individuals and their families is quite huge: patients and parents are facing a complex set of circumstances when they first hear about the term DSD. For this reason, these conditions require a multidisciplinary team, at diagnosis and during each stage of life (11–13). Diagnosis and then follow-up are quite challenging for clinicians, including crucial aspects like sex assignment (and re-assignment in some cases), gonadal management (including the decision of gonadectomy or gonadal surveillance) and pubertal induction (if needed). A correct and preferable early diagnosis could represent a key factor for improving quality of life of patients and their families, followed by a constant psychological support (12, 14, 15).

The most common condition in 46,XX DSD is represented by Congenital Adrenal Hyperplasia (CAH) due to 21-hydroxylase deficiency, with an overall incidence in the Caucasian population of 1:30.000 female newborns (16). Other forms of CAH include 11ß-hydroxylase deficiency, 3ß-hydroxysteroid dehydrogenase type 2 deficiency, and the newest P450 oxidoreductase deficiency, characterized by DSD also often associated with skeletal defects (16).

However, although rarer, other defects involving specific genetic and/or steroidogenesis pathways could determine a DSD condition in 46,XX subjects.

The aim of this review is to summarize the most relevant data on the pathogenesis, diagnosis, clinical and therapeutic management of 46,XX DSD, outside CAH. The first part will focus on sexual differentiation, with a special emphasis on genetic pathways involved in gonadal development, highlighting their role on 46,XX DSD. In the second part the author will discuss clinical management of specific diseases.

## 2 Sexual development in 46,XX

Sexual development is a complex multistep process through which the undifferentiated embryonic structures develop toward a male or female phenotype (7, 8). Until approximately the fifth-sixth week post-fertilization, no sexual differences are observable in a XX or XY fetus. Gonadal determination into either ovaries or testes (or ‘atypical’ gonads in DSD) plays a key role in sexual differentiation, influencing individual’s internal and external genitalia and secondary sexual characteristics (7–10, 17–19).

### 2.1 Gonadal determination

In the last decades there has been a considerable increase in our knowledge of the genes and mechanism involved in the development and function of human gonads.

In 1940s, after Jost’s discovery that the precocious removal of embryonic gonads in rabbits leads to a female phenotype (regardless chromosomal assessment) (20), the ovarian development was long thought to be just a passive or default process. In 1990, the discovery of *SRY* gene (21), also contribute to this “classic theory” for which ovarian development has been considered just as a consequence of the absence of the *SRY*. So, the crucial event in sex determination was whether or not a testis developed. This prompted the researchers to focus on the discovery of genes eventually leading to testis differentiation (22–25).

However, the occurrence of male phenotype in XX individuals in the absence of *SRY* gene, or the cases of XY sex reversal in the presence of *SRY*, led to hypothesize that *SRY* could act as a repressor of the female pathway, also highlighting the hypothesis of the existence of a gene ‘Z’ repressing male development and/or activating female development (26, 27). This model emphasized the theory of the antagonistic nature of male and female specific genetic pathways in the ‘battle of sexes’ during embryonic development (8).

Although to date the precise genetic mechanism of sexual development is still not completely understood, especially in females, it is widely believed that the genetic programs of male and female differentiation are closely intertwined and determined by antagonistic pathways (8, 9, 17, 19).

In humans, the gonadal primordium (also known as the genital ridge) first appears at the 4^th^ week of gestational age, arising from the urogenital ridge comprising of the pronephros (from which the adrenal primordium derives), the mesonephros (from which the gonadal primordium derives) and the metanephros (from which the reno-urinary system derives) (28).

**Figure 1:**
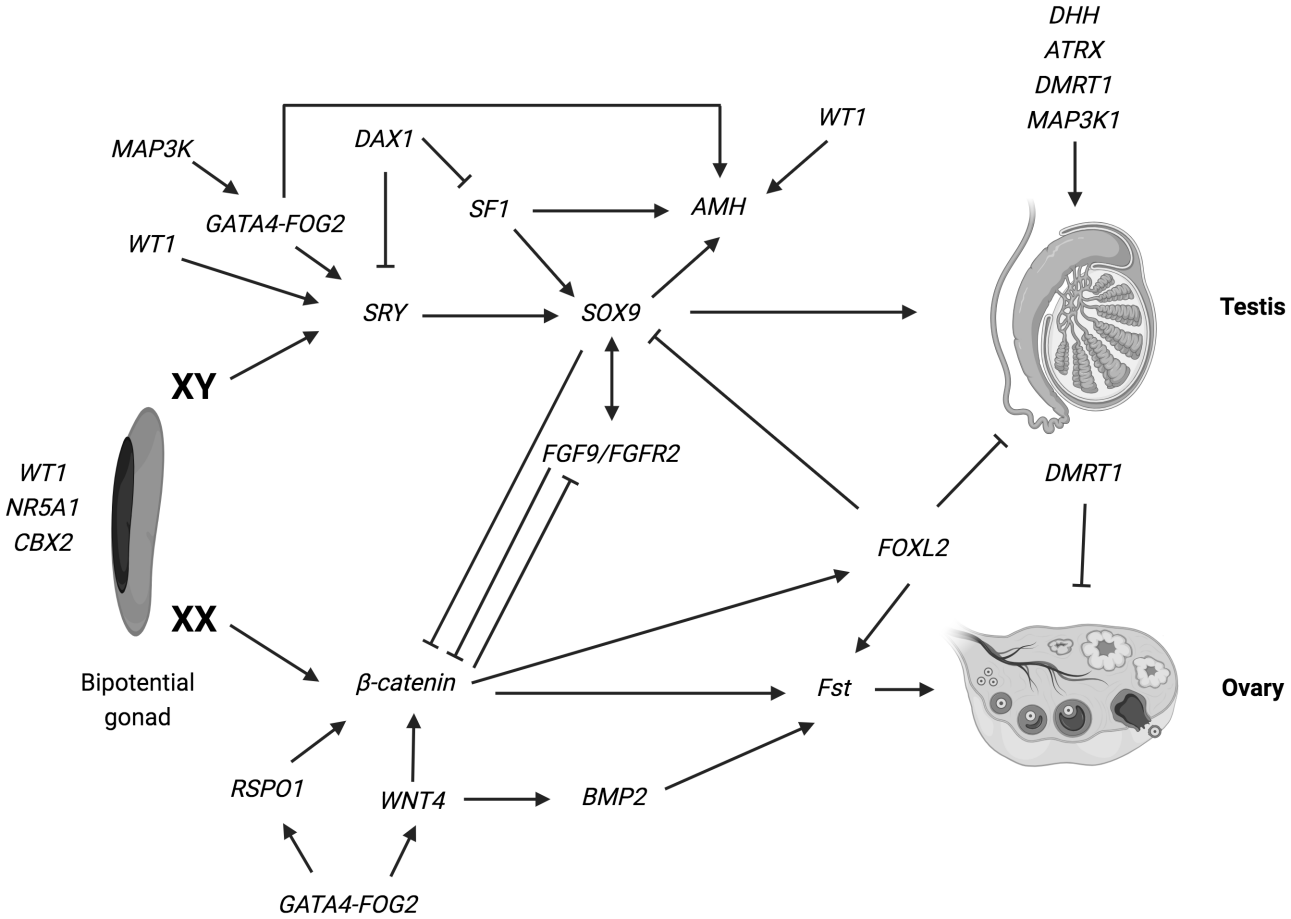
Overview of the genetic pathways involved in gonadal determination. Arrows indicate activation of a downstream target. Lines ending in bars indicate repression of a downstream target. The genes identified as vital for the development of the bi-potential gonad include: nuclear receptor subfamily 5, group A, member 1 (*NR5A1*); Wilms’ tumor suppressor 1 (*WT1*) and chromobox homolog 2 (CBX2) (ref (2, 6, 8–10, 28–30). Various genes have been implicated in the pathways leading the bipotential indifferent gonad either into an ovary or a testis. In XY: *SRY* determine an increase of *Sox9* expression, which then stimulate *Fgf9* expression. Both *Fgf9* and *SOX9* suppress the female specific pathway, especially β-catenin and *WNT4*, supporting testis specific program. Numerous other genes such as *WT1, DAX1, AMH, MAP3K1* and *DMRT1* are necessary for the development and maintenance of testicular gonad. In XX: *SRY* is absent and specific genes are involved in ovarian development: *WNT4* and *RSPO1* have a synergic role on the activation of *β-catenin*, that suppress the *SOX9/Fgf9* testicular pathway. Moreover, *WNT4, RSPO1* and *FOXL2* active *Fst* (follistatin) expression (ref. 17–19). Figure adapted from Tevosian SG. Genetic control of ovarian development. Sex Dev. 2013;7 (1–3):33–45 (ref.^17^); Eggers S, Ohnesorg T, Sinclair A. Genetic regulation of mammalian gonad development. Nat Rev Endocrinol. 2014;10 (11):673–683 (ref.^18^); Ohnesorg T, Vilain E, Sinclair AH. The genetics of disorders of sex development in humans. Sex Dev. 2014;8 (5):262–272 (ref.^19^). Created with BioRender.com.

Since the 90s, there has been a significant increase in our knowledge of the genes required for this early step of sexual development, that is the formation of the bipotential undifferentiated gonad. The genes identified as vital for the initial process of gonadal ridge formation include nuclear receptor subfamily 5, group A, member 1 (*NR5A1*); Wilms’ tumor suppressor 1 (*WT1*); GATA-binding protein 4 (*GATA4*); chromobox homolog 2 (*CBX2*); LIM homeobox gene 9 (*LHX9*); empty-spiracles homeobox gene 2 (*EMX2*) (2, 6, 8–10, 28–30) (**Figure 1**).

Modification of genetic pathways, due to loss of function of the above listed genes, at this early stage of human gestation, may lead to DSD (31–40). In **Table 1** we have summarized current understanding on genes involved in genital ridge development and their pathogenetic role in 46,XX individuals.

**Table 1:** Genes involved in genital ridge development and their pathogenetic role in 46,XX DSD.

Gene	Chr.	Function	Human pathologies	Molecular pathogenesis (ref)
***NR5A1***	9q33.3	Transcription factor/Nuclear receptor	Ovotesticular DSD Testicular DSD Premature ovarian insufficiency	p.Arg92Trp variant (34–38) Loss of function (39)
***WT1***	11p13	Transcription factor	Ovotesticular DSD Testicular DSD Premature ovarian insufficiency	Missense and frameshift variants impacting the 4^th^ ZF (31) Loss of function (32, 33)
***CBX2***	17q25.3	Transcription factor	Gonadal dysgenesis Premature ovarian insufficiency	Loss of function (40)

Until the beginning of differentiation at 6^th^-7^th^ week of gestation, there are no structural differences between XX and XY gonadal primordium (7). In 46,XX fetuses, the gonads remain undifferentiated for a longer period of time compared to 46,XY, probably due to a later expression of the specific ovarian differentiation pathways (9, 17, 30).

The two main roles of the ovary are the production of steroid hormones and the generation of mature oocyte. For these reasons, ovarian differentiation is characterized by two milestones: (i) the entry of XX germ cells into meiosis and (ii) follicle formation (41, 42). Primordial cells differentiate into oogonia from week 8; at week 11-12, germ cells enter into the first meiosis and primordial follicles develop. At week 15, the primary follicles develop, with the differentiation of theca cells. By the time of birth, the majority of the oocytes in the human ovary are contained in primary follicles, and a few in primordial follicles; the number of secondary or tertiary antral follicles is very small (42).

Although the female regulatory cascade still lacks a ‘master’ regulator as an equivalent of *SRY* gene in males, it came to light that specific pathways, involving different genes and transcription factors, such as the wingless-type MMTV integration site family, member 4 (*WNT4*), R-spondin1 (*RSPO1*), Forkhead box L2 (*FOXL2*), *Gata4/6* and *Fog2* and *CTNNB1/beta catenin*, are vital for promoting ovarian differentiation and oocyte maturation and maintenance (8–10).

Components of both male and female pathways antagonize each other to promote development of either testes or ovaries (**Figure 1**).

Changes on these specific pathways, altering this perfect antagonism of ‘battle of sexes’, determine several spectrum of DSD conditions, with variable phenotypes (43). In **Table 2** we have summarized current knowledge on genes involved in gonadal development and their pathogenetic role in 46,XX DSD (41, 44–51).

**Table 2:** Genes involved in gonadal development and their pathogenetic role in 46,XX DSD.

Gene	Chr.	Function	Human pathologies	Molecular pathogenesis (ref)
***SRY***	Yp11.3	Transcription factor	Testicular DSD	Gain of function (44)
***SOX9***	17q24.3	Transcription factor	Testicular DSD and Ovotesticular DSD	Gain of function (45)
***SOX3***	Xq27.1	Transcription factor	Testicular DSD and Ovotesticular DSD	Gain of function (46, 47)
***WNT4***	1p36.12	Signaling protein	Testicular DSD	Loss of function (48, 49)
***RSPO1***	1p34.3	Signaling protein	Testicular DSD	Loss of function (50, 51)
***FOXL2***	3q22.3	Transcription factor	Premature ovarian insufficiency	Loss of function (41)

### 2.2 Internal genitalia differentiation

The internal genitalia are similar in both sexes until 9^th^ week of gestation, when they will differentiate into specific male or female ducts, depending, in turn, by gonadal differentiation. At 5^th^ of gestation, two pairs of ducts, derived from the intermediate mesoderm, begin to develop in fetus: the Wolff ducts, which eventually lead to the epididymis, vas deferens and seminal vesicles) and Mullerian ducts (lateral and parallel to Wolff), which eventually lead to the uterus, fallopian tubes, cervix and the upper third of the vagina (52).

After gonadal differentiation, in case of testis development, synthesis of testosterone by Leydig cells from 9-10^th^ of gestation and anti-mullerian hormone (*AMH*) by Sertoli cells from 12^th^ week of gestation, determine the regression of Mullerian ducts and the stabilization of Wolff ducts. On the contrary, in females, the absence of elevated androgens and *AMH* determine the regression of Wolff ducts and development of Mullerian structures (42, 52).

During the embryogenesis, in XX individuals, a maldevelopment of Mullerian ducts may occur, determining the Mayer-Rokitansky-Küster-Hauser (MRKH) syndrome, that will be discussed later in detail.

### 2.3 External genitalia differentiation

Development of external genitalia depends on androgen-independent and androgen-dependent two different pathways. The first phase begins at 5^th^ week of gestation, with the formation of different structures, identical in both sexes: the genital tubercle, located just cranial to the midline opening of the urogenital ostium; the urogenital folds, laterally to the ostium; and the genital swelling/labioscrotal folds, laterally to the urogenital folds. Formation of these structures occurs identically in males and females, during the so-called ambisexual stage of external genitalia development, a hormone-independent phase, from 5^th^ to 12^th^ week of gestation (53).

After this first phase, in males, the androgen production determines the elongation of genital tubercle to a penis, the development of the penile urethra from the urogenital folds and the fusion of labioscrotal folds in the midline to form the scrotum. In females, the absence of elevated amount of androgens determine that genital tubercle differentiate into the clitoris, the urogenital ostium remains open with the subsequent development of the anterior urethra ostium and posterior vagina ostium, the urogenital folds become the labia minora and the labioscrotal folds become the major labia (53, 54).

In XX individuals, fetuses exposure to androgens between 8^th^ and 15^th^ week of gestation determine virilization of external genital with several degree of ‘masculinization’, as in the cases of 46,XX newborns with atypical genitalia, due to gonadal maldevelopment or steroidogenesis defects. These conditions will be discussed below in detail.

## 3 46,XX Differences of Sex Development

According to the Chicago Consensus (11), recently revised in a European Consensus Statement (12), 46,XX Differences of Sex Development (DSD) include a wide spectrum of conditions due to atypical gonadal development or altered hormonal secretion; in the same group are also classified patients with atypical differentiation of Mullerian structures, affected by the Mayer-Rokitansky-Kuster-Hauser syndrome (MRKH).

Disorders/Differences of gonadal development include testicular DSD, ovotesticular DSD and primary ovarian insufficiency. Disorders/Differences of androgen excess comprise the different forms of Congenital Adrenal Hyperplasia (not discussed in this review), Aromatase deficiency and Maternal causes of androgen excess, usually Luteoma.

In the next paragraphs we will summarize the most relevant data on diagnosis, clinical aspects and management of these rare diseases, outside CAH.

### 3.1 46,XX DSD due to disorders/differences of gonadal development

#### 3.1.1 46,XX testicular DSD

It is characterized by the presence of testes in 46,XX individuals, with concomitant absence of Mullerian derivatives. In 60s A. de la Chapelle identified these subjects as ‘XX males with male phenotype, male psychosexual gender identity, gonads differentiated as testes without macroscopic and microscopic evidence of ovarian tissue and absence of female internal genitalia’ (55).

##### 3.1.1.1 Genetic

The prevalence of this disease is estimated as 1:20.000 (56) and in the majority of cases (almost 90%), the pathogenetic cause is the translocation of *SRY* gene to the X chromosome or more rarely to an autosome (56). 46,XX testicular DSD *SRY* negative, may be due to an increased expression of genetic pathways involved in testicular differentiation and/or an insufficient expression of genetic pathways involved in ovarian differentiation (43) (**Table 2**).

##### 3.1.1.2 Clinical aspects, puberty, sex hormone replacement therapy and possibility of fertility

From a clinical point of view, affected patients usually present with normal virilized male external genitalia; in other cases, newborns may present with atypical genitalia (46–48, 50, 51, 57–63) (**Table 3**). In patients with normal male external genitalia, diagnoses may be delayed until adult life when they could be referred to an endocrinologist because of infertility. Youngest boys may also be referred to a pediatric endocrinologist because of puberty delay or testicular hypoplasia (43) (**Table 3**). It has been speculated that at pubertal age, the absence of Y chromosome and genetic pathway needed for testicular maintenance, determine a progressive germ cell loss, azoospermia and consequent reduced testicular volume (64).

**Table 3:** Genetics, clinical phenotypes and hormonal assessment of *SRY*-negative 46,XX testicular and ovotesticular DSD.

Pathogenesis	Genetic findings	Genital phenotype	Gonadal histology	Hormonal assessment	Sex of rearing	Ref.
**Increased *SOX9* expression**	Duplication of *SOX9* gene	Atypical genitalia Male genitalia	NA Testis	NA FSH, LH, T prepubertal T post HCG test 2.1 ng/ml	NA Male	(59) (60)
	Rearrangement of *SOX9* regulatory regions	Infertility, gynecomastia Atypical genitalia Infertility Mild intellectual disability Atypical genitalia	NA Ovotestis NA NA NA	Hypergonadotropic hypogonadism FSH, LH, T prepubertal T post HCG test 76 ng/dl Hypergonadotropic hypogonadism NA FSH, LH as minipuberty T 113 ng/dl, AMH 19.4 ng/ml	Male NA Male Male Male	(61) (61) (61) (61) (62)
**Increased *SOX3* expression**	Duplication of *SOX3* gene	Atypical genitalia	NA	NA	Male	(47)
	Rearrangement of *SOX3* regulatory regions	Atypical genitalia	Ovotestis	T post HCG test 24.6 nmol/l	Male	(63)
**Increased *SOX10* expression**	Duplication of chr.22q	Hypospadias Atypical genitalia	NA Ovotestis	NA NA	Male NA	(57) (58)
**Decreased *WNT4* expression**	*WNT4* mutation	Atypical genitalia	Testis or ovotestis	NA	NA	(48)
**Decreased** ***RSPO1* expression**	*RSPO1* mutation	Atypical genitalia	Ovotestis	NA	Female	(50)
	*RSPO1* deletion	Atypical genitalia	Gonadal dysgenesis	NA	Male	(51)

NA, Not available; FSH, Follicle-stimulating hormone; LH, Luteinizing hormone; T, Testosterone; HCG test, human chorionic gonadotropin stimulation test.

Adapted from Romina P. Grinspon, Rodolfo A. Rey; Disorders of Sex Development with Testicular Differentiation in SRY-Negative 46,XX Individuals: Clinical and Genetic Aspects. Sex Dev 28 May 2016; 10 (1): 1–11 (ref 43).

In these patients, gender identity is almost always male, but they would need testosterone replacement therapy starting from young adult age (43). It was suggested that levels of testosterone may be normal during adolescence, but decreased in adulthood (65), however, few data have been published so far. Moreover, no studies on treatment to induce or sustain puberty in male patients with gonadal dysgenesis are available in literature (66). Hormone replacement therapy (HRT), if needed, consists of Testosterone treatment, with formulations and therapeutic schemes recommended in International clinical practice guidelines (66–68), that we have summarized in **Table 4**.

**Table 4:** Testosterone replacement therapy in boys and adults with hypogonadism (ref 66-68).

Testosterone [T] formulation	Therapeutic schemes
T enanthate, cypionate or mixture of T esters, i.m. injection	Initial dose: 25-50 mg monthly. Increase of 50 mg every 6-12 months Adult dosage: 150-200 mg every 2 weeks
T undecanoate, i.m. injection	Used for pubertal induction only in young men Adult dosage: 750 mg every 10 weeks 1000 mg every 10-14 weeks
T transdermal gels	Few data available **Gel 1% [Androgel®].** Initial dose: 0.5 g/daily. Increase based on T level: 1.0, 1.5, 2.5, 3.0 or to 5 g/daily as needed Adult dosage: 5.0-10.0 g/daily **Gel 2% [Fortesta®, Tostrex®].** Initial dose: 10 mg/daily Adult dosage: 40-80 mg/daily
T undecanoate, oral tablets	No data available in adolescent population. Adult dosage **[Andriol®]**: 40-80 mg, 2-3 times/day New formulation for adults, approved in USA **[Jatenzo®]**: 158-396 mg twice/daily.
T transdermal patches	Prepubertal 14-16 ys: 2.5 mg over 12 h, overnight Partially virilized 17-19 ys: 2.5 mg/daily Virilized men >20 ys: 5 mg/daily
T pellets subcutaneous	13.9-17.5 ys: 8-10 mg/Kg every 6 months, for 18 months
T intranasal	No data available in adolescent population Adult dosage: 11 mg three times a day
T transbuccal bioadhesive tablets	No data available in adolescent population Adult dosage: 30 mg twice daily

Recently, Chen et al. retrospectively revised a population of 144 males with 46,XX DSD (71 *SRY* positive, 15 *SRY* negative), analyzing clinical characteristics and assisted reproductive technology (ART) outcomes (69). The mean age of patients included in the study was 29.06 ± 4.5 yrs. Greater than 90% of cases had elevated levels of follicle-stimulating hormone and luteinizing hormone, almost 63% of cases had low testosterone values. The mean volume (95% CI) of left and right testis was 2.16 (1-82-2.49) and 2.16 (1.83-2.49) ml, respectively: 143 patients had bilateral atrophic testes with testicular volume less than 15 ml on both sides. Among patients with normal ejaculatory function, azoospermia was found in the totality of cases, all presenting the deletion of *AZFa, AZFb* and *AZFc* regions. Fertility was achieved in 87 patients, through ART: the live birth rates using artificial insemination (AID) or *in vitro* fertilization (IVF) with donor spermatozoa was 18.11 and 58.09% respectively, as already described in other retrospective studies of ART (70).

#### 3.1.2 46,XX Ovotesticular DSD

The differential diagnosis between ovotesticular and testicular DSD is based on histological analysis. It requires the concomitant existence of testicular tissue (seminiferous tubules) and ovarian tissue (follicles containing oocytes) that can be found in each of the two gonads (bilateral ovotestis), just in one of the two gonads (unilateral ovotestis) with the other one normally differentiated as testis or ovary, or one testis in one side and ovary on the other (lateral ovotestis). The most common for is the unilateral (ovotestis/ovary) in 34% of cases, followed by bilateral ovotestis in 29% of cases, lateral (ovary/testis) in 25% of cases and unilateral (ovotestis/testis) in 12% of cases (71).

##### 3.1.2.1 Genetic

Contrary to Testicular DSD, 90% of patients are *SRY* negative. However, similar to testicular DSD, also in these cases, a genetic imbalance of testis and ovarian pathways determining an increased expression of pro-testis genes and an insufficient expression of pro-ovarian/anti-testis genes, results crucial for the onset of these diseases (43, 72) (**Table 3**).

##### 3.1.2.2 Clinical aspects, puberty, sex hormone replacement therapy and possibility of fertility

The clinical presentation is highly variable, ranging from a normal male phenotype or mild/severe hypospadias to a female presentation with genital tubercle hypertrophy. Less virilized patients may also present Mullerian derivatives (46–48, 50, 51, 57–63) (**Table 3**).

Sex assignment represents an important challenge for clinicians, and the multidisciplinary team together with patients and families, will apply clinical and ethical considerations discussed in the Consensus Statement (12), to ensure the best quality of life of each affected individual (14, 73–75). These state that the following principles should guide clinical decisions: minimizing physical and psychosocial risks, preserving the potential for fertility and satisfying sexual relations in adolescence and adulthood, leaving options open for the future if necessary, respecting the parents’ wishes, beliefs and sociocultural tradition, when possible, to guarantee the best options for a healthy life (76) (health is a state of complete physical, mental and social well-being and not merely the absence of disease or infirmity; WHO, 1948).

When the sex may not be easily defined at birth, more time is needed to determine the gender identity and the hormonal assessment of patients, in terms of androgen or estrogen gonadal production, waiting until pubertal age. Patients and families will be followed by a specialized DSD multidisciplinary team at each stage of life (12).

The risk of tumor development in the testicular portion it has been reported to be low, probably due to the absence of Y chromosome (77); the ovarian tissue, before puberty, remain quiescent. For these reasons, in view of the unpredictable gender outcome and in line with the recent recommendations of human rights principles, the irreversible gonadectomy should be avoided in any child with OT- DSD and postponed at pubertal age, after discussion with patients theirselves and families (78).

At the onset of puberty, gonadectomy would be needed, to avoid undesirable gonadal hormone production. However, surgical irreversible procedure should be performed until patient’s gender identity is confirmed by the psychological and psychiatric team. When gender identity is still uncertain, temporary treatment with GnRH analogs has been proposed, becoming a well-established clinical practice in DSD nowadays (78, 79), as for young adolescents with gender dysphoria (80).

Few studies have reported pubertal and fertility outcomes in OT-DSD so far, analyzing just small cohorts (79, 81). Some XX-OT boys may start and develop puberty spontaneously, other may need HRT to induce puberty or maintain testosterone level through adulthood (78, 80). As previously discussed, the absence of Y chromosome and genetic pathway needed for testicular maintenance, generally determine a progressive germ cell loss and azoospermia in male individuals (78). Female OT-DSD patients, who have normally developed ovarian tissue, may have physiological pubertal development and regular cyclic menstruation (77, 78, 80).

Several uneventful pregnancies, spontaneous or induced through ART, have been reported (78, 79, 82–85).

Hormone replacement therapy, if needed, consists of Testosterone or Estrogen treatment, depending on gender assignment, with formulations and therapeutic schemes recommended in International guidelines (66).

As previously discussed, no studies on treatment to induce or sustain puberty in male patients with gonadal dysgenesis are available in literature (66). Therapeutic approaches with Testosterone, standardized in clinical practice, have been summarized in **Table 4**.

On the contrary, looking at clinical studies on treatment to induce or sustain puberty in females with gonadal dysgenesis, several randomized trials and cohort studies have been compared (66); however, the majority of these, have brought patients diagnosed with Turner syndrome, primary ovarian failure or transgender women (66, 86, 87). Further specific studies, focusing on DSD patients, are needed to evaluate the best therapeutic approach ensuring a good quality of life on a long-term follow-up. In **Table 5** we have summarized the most common preparations and relative therapeutic schemes that can be used for pubertal induction and maintenance in DSD girls with hypogonadism.

**Table 5:** Estrogen and progesterone preparations for pubertal induction and maintenance in girls with hypogonadism (ref 66, 85).

Estrogen Preparations	Doses available	Therapeutic schemes
Transdermal E2:		
-Menostar -Vivelle Mini e Vivelle Dot -Estraderm MX -Generic (different brands in different countries) -E2 gel 0.1% -E2 gel 0.06%	14 µg patch 25, 37.5, 50, 75, 100 µg patches 25, 50, 100 µg patches 25, 37.5, 50, 75, 100 µg patches 0.5 and 1 mg E2/sachet 0.75 mgE2/pump	Only used for low dosing: 3-7 ug/d or half path weekly Initial dose: part of patch twice weekly Adult dosage: 50-100 µg twice weekly Not used for pubertal induction Adult dosage: 1-2 sachet daily Not used for pubertal induction Adult dosage: 1-3 pumps daily
E2 oral options:		
-17β-estradiol or Estradiol valerate -Ethinylestradiol (EE2)	0.5, 1, 2, 4 mg	Initial dose: 5 µg/Kg/day (part of a pill daily) Adult dosage: 1-4 mg/day Initial dose: 2 µg/day Adult dosage: 10-20 µg/day

Progesterone Preparations (if uterus present)	Doses available	Therapeutic schemes
Medroxyprogesterone acetate or Dydrogesterone Micronized progesterone	10 mg tablet 100, 200 mg tablet	Give with E2, or alone for 10 days/cycle Give with E2, or alone for 10 days/cycle

Combined E2/Gestagen		
Sequential patch:		
-Climara Pro -Combipatch	E2 0.045 mg/levonorgestrel 0.015 mg/24 h E2 0.045 mg/norethidrone 0.014 or 0.25 mg/24 h	Not used for starting puberty Adult dosage: 1 patch weekly Not used for starting puberty Adult dosage: 1 patch weekly

Sequential pills:		
-Femoston 1/10 or 2/10 -Femoston Continu	Tablet 1-14: 1-2 mg E2; Tablet 15-28: 1-2 mg E2 + 10 mg dydrogesterone All tablets: 1mg E2 + 5 mg dydrogesterone	Not used for starting puberty Adult dosage: 1 tablet/day Not used for starting puberty Adult dosage: 1 tablet/day

#### 3.1.3 Premature ovarian insufficiency (POI)

According to the ESHRE guidelines, Premature ovarian insufficiency (POI) is defined as a clinical syndrome characterized by loss of ovarian activity before the age of 40 years, determining menstrual disturbance (amenorrhea or oligomenorrhea) with raised gonadotrophins and low estradiol (88).

##### 3.1.3.1 Genetic

A wide range of different etiological causes may determine POI disease, including genetic, autoimmune, metabolic, infectious and iatrogenic factors (41, 89).

##### 3.1.3.2 Clinical aspects, puberty, sex hormone replacement therapy and possibility of fertility

The clinical presentation of POI is highly heterogeneous as it can be associated with gonadal dysgenesis and consequent absence of spontaneous pubertal development, primary amenorrhea or secondary amenorrhea due to anticipated depletion of the ovarian reserve before 40 years of age.

Patients with autoimmune or genetic disorders, which it is known to have an increased risk to develop a POI (such as X chromosome defects, *BPES, AIRE* and other), may benefit from fertility preservation at young age with ovarian tissue or egg freezing (90). In the same way, the evidence of pathogenetic variants in adult women already diagnosed with POI, could be extremely useful for female relatives, who can be precociously referred to specialized team of endocrinologists and gynecologists.

Hormone replacement therapy consists of Estrogen and Progesterone treatments, with formulations and therapeutic schemes recommended in International guidelines (66), summarized in **Table 5**.

### 3.2 46,XX DSD due to atypical differentiation of Mullerian structures

#### 3.2.1 Mayer-Rokitansky-Küster-Hauser syndrome

Mayer-Rokitansky-Küster-Hauser (MRKH) syndrome, also referred to as Müllerian agenesis or aplasia, is a congenital disorder caused by embryologic underdevelopment of the Müllerian duct and characterized by agenesis of the uterus, the cervix and the upper two-thirds of the vagina in otherwise phenotypically 46,XX females. The ovaries, considering their different embryogenesis (as mentioned previously), are typically normal in morphology and function (91).

MRKH syndrome has an estimated incidence of about 1:4.000 to 1:5.000 female live births (92) and it is classified among the most severe uterine malformation by the “European Society of Human Reproduction and Embryology” and the “European Society for Gynaecological Endoscopy” classification. It represents the second most common cause of primary amenorrhea after ovarian insufficiency, reported in ~ 16% of female with primary amenorrhea (93).

##### 3.2.1.1 Genetic

The etiology of MRKH syndrome remains unclear. Environmental and genetic causes that may interfere during the embryonic development have been proposed (91, 94). However, currently, the pathogenesis remains not completely understood and the majority of cases do not have a molecular diagnosis. Also, the inheritance is not completely clear: the identification of monozygotic twins discordant for MRKH syndrome (95) and the absence of genital malformations in biological children of women with MRKH syndrome born from surrogate mothers (96), support the sporadic nature of the disease; however, familial cases have been described (97) indicating that, at least in a subgroup of patients, MRKH syndrome may be an inherited disorder.

The most reported genes implicated in the pathogenesis of MRKH syndrome have been *WNT4, LHX1* (LIM homebox protein 1) and *HNF1B* (hepatocyte nuclear factor-1B) (98). In particular, *LHX1* and *HNF1B*, are considered important transcription factors regulating Mullerian ducts development (98). Mutations in *LHX1* (99) and deletions at 17q12, encompassing *LHX1* and *HNF1B*, have been detected in patients with MRKH syndrome (100, 101); mutations in the *HNF1B* gene have been detected in a familial case, in which two out of four female mutation carriers were affected by uterine malformations (102).

##### 3.2.1.2 Clinical aspects, puberty, sex hormone replacement therapy and possibility of fertility

Physical examination of patient with MRKH syndrome reveals female external genitalia with short blind-ending vagina. Patients usually reach puberty at the physiological time, showing normal development of secondary sex characteristics and do not need HRT. The most frequent reason for referral to the endocrinologist or gynecologist is primary amenorrhea, and the median age at first presentation has been reported to be 17.5 years (92). In rare cases, patients may be referred at younger age for ‘incidental’ evidence of uterus agenesis at abdomen ultrasound or abdominal surgery performed for other reasons.

The role of an expert multidisciplinary team (MDT) is essential for the diagnosis and management of the disease.

Pelvic ultrasound (US) is considered the first line diagnostic tool, demonstrating the absence of uterus but the presence of normal bilateral ovaries; pelvic magnetic resonance imaging (MRI) is the gold standard for diagnosis, showing the Müllerian structures in detail, with a better resolution compared to the US (103, 104). One of the most common condition that may be confused with MRKH syndrome is Complete Androgen Insensitivity Syndrome (CAIS), however with a 46,XY karyotype, so the importance of MDT with a great experience on DSD management.

The evaluation of the presence of concomitant congenital extragenital anomalies is essential in patients with MRKH syndrome: in some individuals, uterine maldevelopment may be associated with urological abnormalities or other malformations (91). For this reason, in literature, it is possibly to classify the MRKH syndrome into the type I, (isolated or typical) and type II, where additional extragenital malformation are documented, involving mainly the kidneys and the axial skeleton and less frequently heart and hearing (91).

In European patients with MRKH syndrome, the proportion of type II was reported to be 43.5-54.4% (92, 105), while in Chinese patients was reported to be 30.4% (106). This discrepancy may be explained by ethnic differences. Renal malformations are the most frequent extragenital abnormalities, occurring in ~ 30–40% in European cohorts (92, 105). Unilateral renal agenesis is the most frequent anomaly accounting about half of all renal malformations associated with MRKH syndrome; it is often associated with complete absence of the ipsilateral Müllerian duct which suggests a close relationship between early kidney and Müllerian duct development. Other renal malformations include pelvic kidney, duplex kidney, and horseshoe kidney (92).

Anomalies of the skeleton are the second most frequent extragenital manifestations affecting about ~10–40% of patients (92). Skeletal anomalies typically involve the axial skeleton (scoliosis, Klippel-Feil anomaly, hemivertebrae, rib aplasia) and more rarely the extremities.

Cardiac abnormalities are reported in < 5% of patients (pulmonary valve stenosis, atrial septal defect) (92). Hearing impairment, including both sensorineural and conductive hearing, is generally reported in < 5% of patient, but is not routinely examined (92).

The coexistence of Müllerian aplasia with unilateral renal aplasia/ectopic kidney and cervicothoracic somite dysplasia is called Müllerian aplasia, renal aplasia, and cervicothoracic somite dysplasia syndrome (MURCS) (107).

Caring of patients with MRKH syndrome require a multidisciplinary team consisting of expert gynecologists, surgeons, physiologists and sexologists, playing a key role at diagnosis and long-term follow-up (12).

The creation of a new functional neovagina represents one of the most important intervention to discuss with patients and families. In the last decades, different surgical and non-surgical treatment strategies have been suggested for vaginal reconstruction.

The American College of Obstetricians and Gynecologists (ACOG) has recommended self-dilation therapy using vaginal dilatators as first line approach in most patients, based on the high overall success rate, safeness with low complication rate and reduced operating costs than surgery (108). Adverse effects reported with dilation include urinary complaints, bleeding and pain. Patients should receive a psychological support and encouraged to start dilation when she feels emotionally and physically ready (109).

ACOG recommend that surgery should be reserved for those patients experiencing failure with dilation, choosing among different surgical approaches and techniques. A comprehensive literature review on the management of vaginal agenesis has been conducted by Callens et al. discussing outcome, advantages and disadvantages of the different procedures (110). However, discuss with patients and families the possibility of different therapeutical approaches is absolutely important, highlighting that also the surgical option require a postoperative dilation to ensure a satisfactory long-term outcome.

Currently, patients affected by MRKH syndrome may experience motherhood adopting a child or, in some Countries also with a surrogate pregnancy, transferring the embryo derived from their own oocytes and partner’s sperm (111, 112); very recently, the uterus transplantation technique has also been reported (113). Since the first live birth after uterus transplantation achieved by the Swedish team (113), more than 80 procedures have been performed around the globe and at least 40 children were born (114).

Despite recent scientific progress, further studies are needed to improve quality of life of these patients and reduce psychological discomforts (115, 116).

### 3.3 46,XX DSD due to androgen excess

#### 3.3.1 Aromatase deficiency

Aromatase deficiency (AroD) is a rare genetic condition that is caused by mutations in the *CYP19A1* gene, located on the long arm of chromosome 15 (15q21.1) (16). It is an autosomal recessive disorder, first described by Shozu et al. in 1991 (117); then, almost 40 cases have been described, due to several pathogenetic variations in the *CYP19A1* gene (118).

Aromatase is the enzyme that catalyzes the synthesis of estrogens from androgens. The three main precursors are androstenedione, testosterone, and 16-α-hydroxy dehydroepiandrosterone sulfate, catalyzed into estrone, estradiol, and estriol, respectively. During pregnancy, 16OH-dehydroepiandrosterone sulfate (16OH-DHEAS) arising from fetal liver hydroxylation of fetal adrenal DHEAS represents an important substrate for placental aromatase and subsequent estriol production (119).

##### 3.3.1.1 Genetic

In human, the *CYP19A1* gene and its product aromatase are expressed in the ovary, testis, placenta, adipose tissue, skin, and the brain. The size of the aromatase gene is greater than 123 kb, and its tissue-specific expression is regulated by the use of tissue-specific promoters involving alternative splicing (120). Moreover, *CYP19* gene expression is regulated by several hormones and factors that differ markedly between tissues. Thus, a strict control over tissue-specific expression is needed for proper regulation of estrogen synthesis during fetal development and post-natal life. The biological importance of the aromatase complex is related not only to its role in the synthesis of estrogens, but also to its potential influence on the balance of the androgen-estrogen ratio in different tissues (118).

##### 3.3.1.2 Clinical aspects, puberty, sex hormone replacement therapy

Clinical phenotype in patients affected by AroD is quite variable, depending on the enzymatic activity (121). In most 46,XX newborn diagnosed with aromatase deficiency, atypical genitalia with various degrees of masculinization of the external genitalia have been described (16, 118, 121). A typical aspect that could alert to the possibility of diagnosis is the maternal virilization during gestation, that progressively disappear after delivery.

During infancy and childhood, there are usually no symptoms of aromatase deficiency. However, it has been reported that basal and stimulated gonadotropin levels (LH, FSH) remain significantly high during the infancy and childhood period, resulting in a higher risk of follicular ovarian cysts (122, 123). It has been speculated that a prolonged effect of androgens or lack of estrogens during gestation, persisting into infancy, might result in an irreversible incorrect maturation of the GnRH pulse generator (123).

In adolescent girls, AroD may determine several conditions such as puberty delay, hypergonadotropic hypogonadism or primary amenorrhea, due to estrogen deficiency; moreover, clinical signs of hyperandrogenism may also be present (16). In affected individuals, regular follow-up is needed to avoid long-term consequences of hypoestrogenism.

To date there is no consensus on the appropriate age, type and dosage of estrogen replacement therapy and the usefulness of starting low-dose estrogen treatment from infancy in affected females.

Further retrospective and prospective studies on large population are needed to define the best management of these patients.

## 4 Conclusions

The 46,XX DSD group includes a wide spectrum of conditions, with different etiopathogenesis. Even if the majority of cases are caused by CAH, it is crucial to know that other rarer disorders exist, in order to make a correct and preferable early diagnosis. Patients should be referred to a specialized Centre, where a trained multidisciplinary team (MDT) could manage these children/adolescents (and families), from diagnosis to adulthood. Support group may have a key role, together with MDT, helping families to not fell alone, sharing parents’ and patients’ experiences. While many patients fare well and have a good quality of life, other individuals have expressed a sense of anxiety and discomfort about the DSD condition or have reported poor quality of life. For these reasons is important to improve our knowledge on diagnosis, management and long-term prognosis of these individuals, organizing international collaborative studies focusing on more debate aspects, such as clinical care, psychosocial development and psychosocial adaptation.

## Author contributions

MS: Conceptualization, Writing – original draft, Writing – review & editing. SM: Writing – original draft. CB: Writing – original draft. GR: Writing – review & editing.
